# Mediating effect of vascular risk factors underlying the link between gestational diabetes and cardiovascular disease

**DOI:** 10.1186/s12916-022-02581-0

**Published:** 2022-11-04

**Authors:** Ravi Retnakaran, Baiju R. Shah

**Affiliations:** 1 Leadership Sinai Centre for Diabetes, Mount Sinai Hospital, Toronto, Canada; 2 Lunenfeld-Tanenbaum Research Institute, Mount Sinai Hospital, Toronto, Canada; 3 Division of Endocrinology, University of Toronto, Toronto, Canada; 4 Institute for Clinical and Evaluative Sciences, Toronto, Canada; 5 Department of Medicine, Sunnybrook Health Sciences Centre, 2075 Bayview Avenue, Room G106, Toronto, ON M4N 3M5 Canada; 6 Institute for Health Policy Management and Evaluation, University of Toronto, Toronto, Canada

**Keywords:** Gestational diabetes, Cardiovascular disease, Risk factors, Glycemia, Lipids, Mediation analysis

## Abstract

**Background:**

Women with gestational diabetes (GDM) have an elevated lifetime incidence of cardiovascular disease (CVD), but the basis of this excess risk remains to be established. In this context, we hypothesized that chronic exposure to adverse cardiovascular risk factors may contribute to their elevated risk of CVD. We thus sought to quantify the determinants of CVD risk in women with a history of GDM by performing mediation analyses.

**Methods:**

Women in Ontario, Canada, with a live-birth pregnancy between Jan 1998 and Dec 2017 (*n*=757,541) were followed for a median of 13.2 years and stratified into the following 4 groups: women with GDM who developed CVD (GDM+/CVD+); women without GDM who developed CVD (GDM−/CVD+); those with GDM but no CVD (GDM+/CVD−); and those with neither GDM nor CVD (GDM−/CVD−). Lipids (total cholesterol, LDL, HDL, triglycerides) and glycemic variables (A1c, fasting glucose) were measured between 4.3±3.0 and 4.8±3.4 times over follow-up.

**Results:**

On successive measurements at a median of 4.8, 7.1, and 8.7 years postpartum, respectively, each lipid and glycemic measure progressively worsened from GDM−/CVD− to GDM+/CVD− to GDM−/CVD+ to GDM+/CVD+ (all *p*<0.0001). At each point in time, each of the lipid and glycemic measures was significantly worse in GDM+/CVD+ compared to GDM+/CVD− (all *p*<0.001). Moreover, among women who did not develop CVD, all lipid and glycemic measures were significantly worse in those with previous GDM (all *p*<0.001 for GDM+/CVD− vs GDM−/CVD−). Mediation analyses revealed that the dominant determinants of CVD risk in women with GDM were A1c (56.0% mediation, 95%CI 47.4–67.8) and fasting glucose (47.4%, 38.8–60.8), followed by HDL (25.2%, 21.3–30.7) and triglycerides (12.1%, 9.7–15.6). Upon exclusion of those who developed diabetes during follow-up, the key determinants were HDL (40.8%), fasting glucose (37.7%), A1c (28.6%), triglycerides (21.0%), and LDL (9.9%).

**Conclusions:**

Adverse glycemic and lipid measures mediate the elevated risk of CVD in women with previous GDM, with the impact of lipids particularly evident in those who do not develop diabetes. These findings thus identify potential targets for risk factor monitoring and ultimately early intervention towards the goal of primary prevention of CVD in this at-risk patient population.

**Supplementary Information:**

The online version contains supplementary material available at 10.1186/s12916-022-02581-0.

## Background

It is well established that women who are diagnosed with gestational diabetes mellitus (GDM) have a markedly elevated lifetime risk of progressing to type 2 diabetes (T2DM), with meta-analyses quantifying this risk as 7- to 10-fold higher than that of their peers [1, 2]. In the past decade, a series of studies have demonstrated that women with GDM also have an elevated risk of developing cardiovascular disease (CVD), including both atherosclerotic vascular disease and heart failure [3–10]. Indeed, a recent meta-analysis involving >5.3 million women demonstrated a 2-fold higher risk of CVD in women with previous GDM that emerges within the first decade after the pregnancy [3]. Moreover, when restricted to women who did not develop T2DM, this meta-analysis revealed that GDM was still associated with a 56% higher risk of subsequent CVD events [3]. Thus, the risk of CVD in women with previous GDM is not entirely attributable to intercurrent T2DM, thereby raising the question of the relevant risk determinants in this patient population. The importance of addressing this question is further underscored by the recognition that elucidation of these underlying determinants could enable early intervention aimed at risk modification [11].

It has been known for over 2 decades that women with a history of previous GDM have a greater burden of cardiovascular risk factors than their peers, including higher prevalence rates of dysglycemia, dyslipidemia, and metabolic syndrome [12–15]. Recently, it has emerged that these differences arise over time because of distinct trajectories of rising glycemia and worsening lipids that are present even before the pregnancy in which GDM is diagnosed [16], and that continues in the years thereafter [17]. Indeed, from a life course perspective, the pregnancy can be viewed as a life event that is superimposed upon these pre-existing trajectories of cardiometabolic risk and thereby enables the identification of a patient population (i.e., women with GDM) that is on a high-risk track for future CVD. In this context, we hypothesized that chronic exposure to these glycemic and lipid factors may contribute to their elevated future risk of CVD. If so, it follows that, among women with GDM, the subset that goes on to develop CVD may have a more adverse cardiovascular risk factor profile than their peers. In this study, we thus sought to test these hypotheses and perform mediation analyses to identify the determinants of CVD risk in women with GDM.

## Methods

### Study design and data sources

A retrospective cohort study was conducted using administrative and clinical data at a population level in Ontario, Canada. We used hospital and demographic databases, and a derived database linking delivering mothers with their infants. Laboratory testing results were ascertained from the Ontario Laboratory Information System (OLIS), which has gradually enrolled hospital and community laboratories since 2007. These databases were linked using unique encoded identifiers and analyzed at ICES (formerly the Institute for Clinical Evaluative Sciences). The use of the data in this study was authorized under section 45 of the *Personal Health Information Protection Act, Ontario*, which does not require research ethics board review.

### Population

**Fig. 1:**
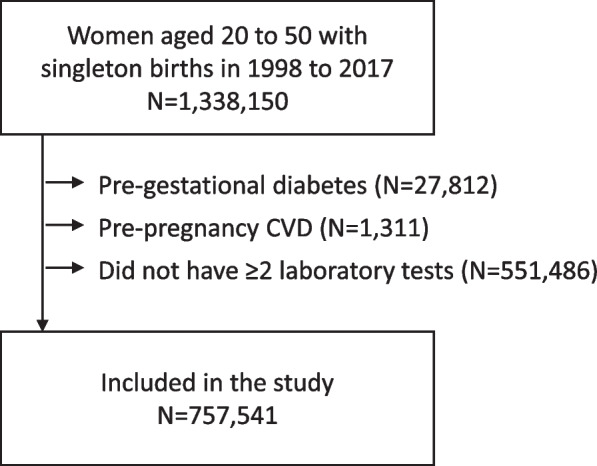
Derivation of the study population

This study included all women between 20 and 50 years of age who had live singleton births between January 1998 and December 2017. Figure 1 shows the derivation of the study population. Women with pregestational diabetes were excluded. For women with multiple pregnancies, we selected the last pregnancy. The index date for each woman was the latest of January 1, 2008 (since OLIS began in 2007), or 30 days after delivery. Women who had a history of CVD hospitalization prior to the index date were excluded (with CVD identified by acute myocardial infarction, acute coronary syndrome, ischemic stroke, transient ischemic attack, coronary revascularization, and carotid revascularization—Table 1 shows the list of codes). All available laboratory test results for six cardiovascular risk factors (total cholesterol, LDL cholesterol, HDL cholesterol, triglycerides, A1c, and fasting glucose) were identified between index and December 31, 2019. Women who did not have ≥2 results for one or more of these risk factors were excluded.

**Table 1:** Codes for defining cardiovascular disease hospitalizations

Acute myocardial infarction	International Classification of Disease, 9th Rev: 410 International Classification of Disease, 10th Rev: I21, I22
Acute coronary syndrome	International Classification of Disease, 9th Rev: 411 International Classification of Disease, 10th Rev: I20
Ischemic stroke	International Classification of Disease, 9th Rev: 434, 436 International Classification of Disease, 10th Rev: I63 (except I63.6), I64
Transient ischemic attack	International Classification of Disease, 9th Rev: 435 International Classification of Disease, 10th Rev: G45 (except G45.4)
Coronary revascularization	Canadian Classification of Procedures: 48.02, 48.03, 48.1 Canadian Classification of Health Interventions: 1IJ50, 1IJ57, 1IJ76
Carotid revascularization	Canadian Classification of Procedures: 50.12 Canadian Classification of Health Interventions: 1JE57

### Exposure and outcomes

The exposure of interest was GDM in the index pregnancy. GDM was ascertained based on diagnosis codes recorded on the delivery hospitalization record. Standard obstetrical care in Ontario includes a 2-step GDM screening strategy consisting of a screening glucose challenge test (GCT) between 24 and 28 weeks’ gestation in all women, followed by a diagnostic oral glucose tolerance test in those in whom the GCT was abnormal [18]. The outcome was the occurrence of CVD hospitalization (see Table 1) occurring between the index date and December 31, 2019.

### Statistical analyses

The women comprising the study population were stratified into four exposure groups based on two features: (i) the presence or absence of GDM and (ii) the presence or absence of hospitalization for CVD. Baseline characteristics of age, neighborhood-level median household income (in quintiles), rurality [19], race [20], and history of prior GDM were compared between the four groups using one-way ANOVA (for continuous variables) and chi-square tests (for categorical variables). In each group, the mean value for each of the first three measurements of each cardiovascular risk factor was calculated, adjusting for age, income, rurality, and race. Mediation analysis was performed to determine the impact of each cardiovascular risk factor on the relationship of GDM on CVD. Using logistic regression with CVD as the dependent variable, the beta-estimates for GDM were compared between a model adjusting for age, income, rurality, race, and prior GDM and a model adjusting for age, income, rurality, race, prior GDM, and the mean value during follow-up of each cardiovascular risk factor. Only women who had ≥2 values for a particular cardiovascular risk factor were included in the analysis of that risk factor, and laboratory tests occurring after a CVD event were not included. Confidence intervals for mediation were determined using bootstrapping with 2000 random samples. Mediation analyses were repeated with the exclusion of women who developed diabetes during the follow-up years after the pregnancy. The rationale for doing so was to determine if mediators of cardiovascular risk differed depending on whether women with previous GDM progressed to diabetes (given its high incidence in this patient population). Diabetes was ascertained from the healthcare claims data using a validated algorithm [21]. P-values <0.05 were considered statistically significant; because of the exploratory nature of the study, we did not adjust for multiple comparisons. Analyses were conducted using SAS version 9.4 (Cary, NC).

## Results

The study population of 757,541 women was followed for median 13.2 (IQR 8.6 to 17.6) years after pregnancy, during which time 2725 women developed CVD and 54,464 developed diabetes. The mean age at diagnosis of diabetes was 42±7 years and the mean age at CVD event was 47±7 years. Table 2 shows the clinical characteristics of the study population stratified into the following 4 groups based on whether or not the women had GDM and whether or not they developed CVD: (i) those with neither GDM nor CVD (GDM−/CVD−; *n*=697,305); (ii) women with GDM who did not have subsequent CVD hospitalization (GDM+/CVD−; *n*=57,511); (iii) those who did not have GDM but had CVD hospitalization (GDM−/CVD+; *n*=2,322); and (iv) women who had both GDM and subsequent CVD hospitalization (GDM+/CVD+; *n*=403). These groups differed in age, race, income, and rurality (each *p*<0.001). In particular, women with either GDM or CVD were more likely to have lower income. South Asian background was more prevalent in women with GDM, whereas women who developed CVD were older and more likely to have rural residency. Median (interquartile range) follow-up from pregnancy was 13.3 (8.8–17.6) years in the GDM–/CVD– group, 11.7 (6.6–18.1) years in the GDM+/CVD− group, 13.8 (9.8–16.9) years in the GDM–/CVD+ group, and 14.1 (10.6–17.2) years in the GDM+/CVD+ group.

**Table 2:** Baseline characteristics of the study population stratified into the following 4 groups: (i) women who had neither GDM nor CVD (GDM− CVD−); (ii) women who had GDM but did not develop CVD (GDM+ CVD−); (iii) women who did not have GDM but developed CVD (GDM− CVD+); and (iv) women who had GDM and developed CVD (GDM+ CVD+)

	GDM− CVD−	GDM+ CVD−	GDM− CVD+	GDM+ CVD+	
Characteristics	***N***=697,305	***N***=57,511	***N***=2322	***N***=403	***P***
Age (years)	39.4 ± 6.5	39.5 ± 6.5	42.3 ± 6.3	42.7 ± 6.2	<0.001
Race *n* (%)					<0.001
Chinese	42,514 (6.1%)	4,726 (8.2%)	43 (1.9%)	≤5 (≤1.2%)	
South Asian	37,043 (5.3%)	4,955 (8.6%)	69 (3.0%)	32 (7.9%)	
General population	617,748 (88.6%)	47,830 (83.2%)	2210 (95.2%)	≥366 (≥90.8%)	
Income quintile n(%)					
Lowest	115,204 (16.5%)	12,720 (22.1%)	511 (22.0%)	113 (28.0%)	<0.001
Second	124,554 (17.9%)	11,692 (20.3%)	493 (21.2%)	82 (20.3%)	
Third	143,552 (20.6%)	11,949 (20.8%)	464 (20.0%)	81 (20.1%)	
Fourth	160,449 (23.0%)	11,752 (20.4%)	481 (20.7%)	79 (19.6%)	
Highest	151,961 (21.8%)	9,232 (16.1%)	364 (15.7%)	43 (10.7%)	
Missing	1585 (0.2%)	166 (0.3%)	9 (0.4%)	5 (1.2%)	
Rurality *n* (%)					
Urban	544,808 (78.1%)	47,447 (82.5%)	1660 (71.5%)	302 (74.9%)	<0.001
Semi-urban	115,160 (16.5%)	7394 (12.9%)	492 (21.2%)	69 (17.1%)	
Rural	37,337 (5.4%)	2670 (4.6%)	170 (7.3%)	32 (7.9%)	
Prior GDM	8075 (1.2%)	7960 (13.8%)	26 (1.1%)	41 (10.2%)	
Developed diabetes during follow-up *n* (%)	37,741 (5.4%)	16,029 (27.9%)	444 (19.1%)	250 (62.0%)	<0.001

Age presented as mean ± standard deviation. Categorical variables presented as *n* followed by percentage in parentheses. Index date was the latest of January 1, 2008, or 30 days after delivery

As would be anticipated, the development of diabetes during follow-up differed between the groups, with the highest incidence in the GDM+/CVD+ group (62.0%), followed by GDM+/CVD− (27.9%), GDM−/CVD+ (19.1%), and GDM−/CVD− (5.4%) (Table 2). Similarly, as expected, the CVD event rate per 10,000 person-years was higher in women who had GDM than in those who did not have GDM (5.8 vs 2.5, *p*<0.0001). Among women who developed diabetes during follow-up, this CVD event rate was higher in those with previous GDM than in those without GDM (11.3 vs 7.4, *p*<0.0001). Similarly, among women who did not develop diabetes, the CVD event rate was higher is those with a history of GDM than in those without previous GDM (3.2 vs 2.2, *p*<0.0001). These event rates indicate that consistent with the literature [3, 10], the development of diabetes is a major contributor to the higher risk of CVD in women with a history of GDM but does not account for all of their elevated risk.

**Fig. 2:**
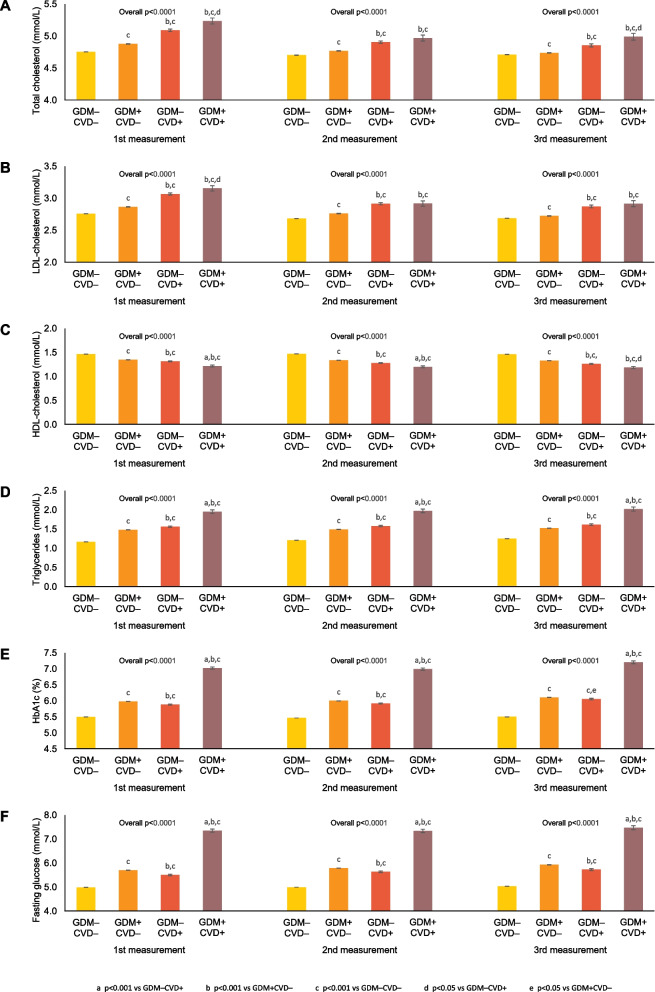
Adjusted mean concentrations of **A** total cholesterol, **B** LDL cholesterol, **C** HDL cholesterol, **D** triglycerides, **E** A1c, and **F** fasting glucose at first, second and third measurements after pregnancy, adjusted for age, race, income, and rurality

Over the course of follow-up, the women underwent measurement of lipids (total cholesterol/LDL/HDL/triglycerides) and glycemic variables (A1c/fasting glucose) between 4.3±3.0 to 4.8±3.4 times. The first 3 tests were performed at a median of 4.8 (IQR 1.8 to 8.9), 7.1 (IQR 3.6 to 11.1), and 8.7 (IQR 5.1 to 12.7) years after the pregnancy. The correlation between the tests is shown in Additional file 1: Table S1. On each of these 3 tests, we evaluated mean adjusted levels of these cardiovascular risk factors in the 4 groups, after adjustment for age, race, income, and rurality. As shown in Fig. 2, total cholesterol (Panel A), LDL cholesterol (Panel B), and triglycerides (Panel D) rose across the 4 groups from GDM−/CVD− to GDM+/CVD− to GDM−/CVD+ to GDM+/CVD+ at each of the 3 points in time (all *p*<0.0001). This pattern was mirrored by a concomitant stepwise decrease in HDL (all *p*<0.0001) (Panel C). Similarly, the glycemic measures of A1c (Panel E) and fasting glucose (Panel F) also exhibited a worsening across the 4 groups at each point in time. Moreover, the pairwise comparisons between groups yielded two other consistent patterns of note. First, at each point in time, each of the lipid and glycemic measures was significantly worse in GDM+/CVD+ compared to GDM+/CVD− (all *p*<0.001). Second, among women who did not develop CVD, all lipid and glycemic measures were significantly worse in those with previous GDM (all *p*<0.001 for GDM+/CVD− vs GDM−/CVD−).

**Fig. 3:**
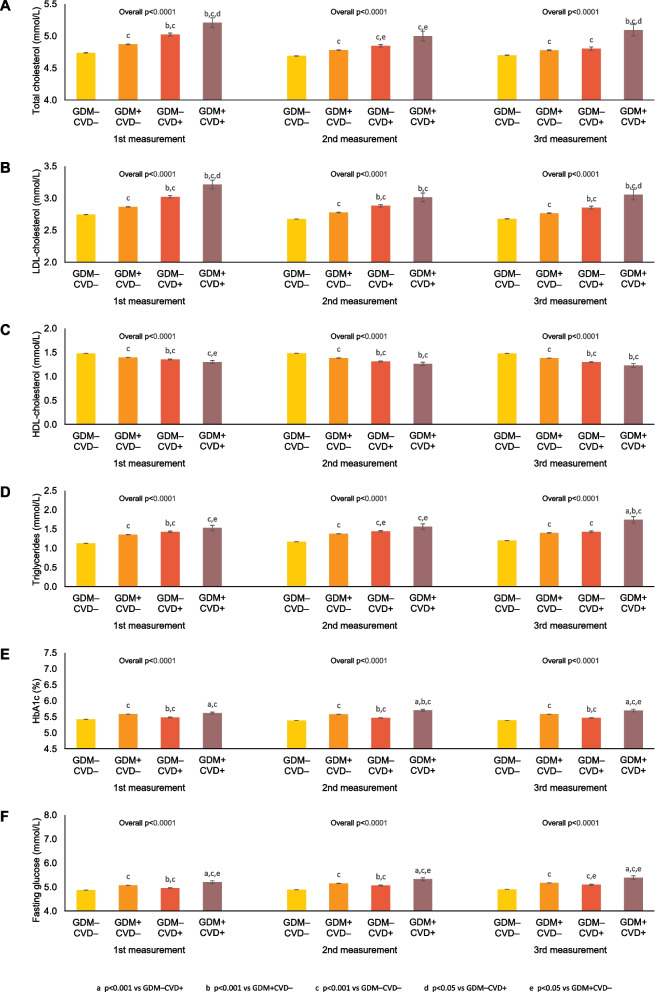
Adjusted mean concentrations of **A** total cholesterol, **B** LDL cholesterol, **C** HDL cholesterol, **D** triglycerides, **E** A1c, and **F** fasting glucose at first, second and third measurements after pregnancy, adjusted for age, race, income, and rurality, after exclusion of women who developed diabetes during follow-up

In the GDM+/CVD+ group, the mean adjusted levels of A1c and fasting glucose were in the diabetes range (≥6.5% and ≥7.0 mmol/L, respectively), suggesting that the intercurrent development of diabetes was a likely contributor to the risk of CVD. Accordingly, we repeated the comparisons of mean adjusted levels of the cardiovascular risk factors between the 4 groups, after excluding the 55,264 women who developed diabetes during follow-up (Fig. 3). Notably, all patterns observed in the full study population (Fig. 2) were maintained when excluding women who developed diabetes (Fig. 3). These preserved patterns included the significant differences in pairwise comparisons between groups. Indeed, each lipid and glycemic measure remained significantly worse in (i) GDM+/CVD+ vs GDM+/CVD− and in (ii) GDM+/CVD− vs GDM−/CVD−). Thus, the worsening of cardiovascular risk factors from GDM−/CVD− to GDM+/CVD− to GDM−/CVD+ to GDM+/CVD+ was consistently observed at each point in time and was not dependent upon the development of diabetes.

Finally, we performed mediation analyses to identify the determinants of CVD risk (Table 3). These analyses revealed that the dominant determinants of CVD risk in women with GDM were A1c (56.0% mediation, 95%CI 47.4–67.8) and fasting glucose (47.4%, 38.8-60.8), followed by HDL (25.2%, 21.3–30.7) and triglycerides (12.1%, 9.7–15.6). Upon exclusion of women who developed diabetes during follow-up, the key determinants were HDL (40.8%), fasting glucose (37.7%), A1c (28.6%), triglycerides (21.0%), and LDL (9.9%). Thus, when women who developed diabetes were excluded, the impact of A1c on CVD risk was reduced (56.0% ➔ 28.6%), as was that of fasting glucose (47.4% ➔ 37.7%). Conversely, the impact of lipid measures increased considerably (HDL 25.2% ➔ 40.8%; triglycerides 12.1% ➔21.0%: LDL cholesterol 0.0% ➔ 9.9%).

**Table 3:** Mediation analysis showing percentage contribution of CV risk factors to the association between GDM and CVD in two settings: (i) all women and (ii) excluding women who developed diabetes

CV risk factor	All women (% mediation)	Excluding women who developed DM (% mediation)
Total cholesterol	− 0.1% (− 0.6–0.3)	4.7% (2.2–14.4)
LDL cholesterol	0.0% (− 0.6–0.6)	9.9% (5.6–30.5)
HDL cholesterol	25.2% (21.3–30.7)	40.8% (24.7–120.1)
Triglycerides	12.1% (9.7–15.6)	21.0% (11.7–73.8)
A1c	56.0% (47.4–67.8)	28.6% (16.1–74.3)
Fasting glucose	47.4% (38.8–60.8)	37.7% (20.3–152.3)

The mediation analysis shows how much the relationship between GDM and CVD is changed by adding the mean value for each cardiovascular risk factor to an adjusted model. For example, adjusting for mean total cholesterol had a negligible effect on the relationship between GDM and CVD, whereas adjustment for mean HDL cholesterol had a much greater impact. Since each risk factor was tested independently of the others, the % mediation can add up to more than 100%. Data are presented as estimated percentage mediation followed by 95% confidence interval in parentheses

## Discussion

This study yields 2 key findings. First, among women with GDM, repeated assessments at a median of 4.8, 7.1, and 8.7 years after the index pregnancy show that those who go on to develop CVD consistently display a more adverse cardiovascular risk factor profile than that of their peers, resulting in greater cumulative exposure to glycemia and dyslipidemia. Of note, these differences persist (but with magnitude attenuated) in the absence of intercurrent progression to diabetes. Second, mediation analyses revealed that these adverse glycemic and lipid measures mediate the elevated risk of CVD in women with previous GDM, with the impact of lipids amplified in those who do not develop diabetes.

Previous studies of the determinants of CVD in women with GDM have understandably focused on the potential impact of diabetes, recognizing the 7- to 10-fold higher risk of T2DM in this patient population [1, 2]. In this context, a meta-analysis of these studies showed that women with GDM have an elevated incidence of CVD even if they don’t develop T2DM [3]. Moreover, a recent study found that intercurrent T2DM explained 23% of the association between GDM and subsequent CVD [10]. Thus, the evidence to date has implicated diabetes as a major contributor but not the sole determinant of CVD risk in this setting [3, 10]. Of note, in the current study, the CVD event rates per 10,000 person-years when stratifying the study population according to presence/absence of GDM and presence/absence of subsequent diabetes are entirely consistent with this concept.

The current study extends the literature by directly evaluating the mediation provided by specific glycemic and lipid variables measured between 4.3±3.0 to 4.8±3.4 times over a median of 13.2 (IQR 8.6 to 17.6) years follow-up. With this approach, we show that women with a history of GDM have greater cumulative exposure to glycemia and dyslipidemia even in the absence of progression to diabetes (Figs. 2 and 3), thereby yielding two implications. First, these data suggest that the adverse risk factor profile should be recognized as a feature of the chronic metabolic dysfunction that characterizes GDM, irrespective of T2DM. Second, the cumulative exposure that is evident upon repeated measurement may be consistent with the hypothesis that the indicated glycemic and lipid variables contribute to the elevated CVD risk in women with GDM, although the observational design of this study precludes inference on causality from the observed associations.

We thus directly addressed the latter hypothesis through mediation analyses, which yielded three additional insights (besides quantifying the relative impact of the specific glycemic and lipid variables). First, the contribution of T2DM to CVD risk in women with previous GDM is supported by (i) the dominant mediating effects of A1c and fasting glucose and (ii) the impact of HDL and triglycerides (i.e., reflecting the dyslipidemic pattern that is typically associated with diabetes). Second, the higher fasting glucose of women with previous GDM holds vascular implications even if they don’t progress to T2DM, as evidenced by the observed persistence of its impact after the exclusion of those who developed diabetes (i.e., 47.4% mediation vs 37.7%). Indeed, the same conclusion applies to A1c, recognizing that, while diminished (from 56.0%), the absolute magnitude of its mediation remained considerable upon exclusion of those with diabetes (at 28.6%). Thus, glycemic exposure below the diabetes diagnostic range contributes to CVD risk in women with GDM, which is particularly noteworthy when one considers their pre-existing trajectories of rising A1c and fasting glucose over time [16, 17]. Third, in women with GDM who don’t develop T2DM, all of the lipid variables (HDL, triglycerides, LDL) show an enhanced impact on CVD risk, again implicating the potential importance of their previously reported trajectories over time [16, 17].

Collectively, these insights hold clinical implications that may be relevant to the management of CVD risk in women with a history of GDM. First, these data support the importance of ongoing clinical surveillance for progression to T2DM in this patient population, given its cardiovascular implications. Second, even if they do not develop T2DM, women with previous GDM likely warrant monitoring of glycemic variables and lipids since these factors have now been linked to their future risk of CVD (as per the mediation analysis). Indeed, the pairwise comparisons in Figs. 2 and 3 show that women with previous GDM but no CVD consistently had more adverse glycemic and lipid measures than those with neither GDM nor CVD, thereby raising the possibility that vascular disease may yet arise in the future with the passage of time and longer exposure. Third, the association of glycemic and dyslipidemic exposure with CVD risk following GDM underscores the need for future studies to determine whether risk modification may be achieved in this patient population through early intervention addressing these factors. For example, while we are currently conducting a randomized placebo-controlled trial to determine whether the sodium-glucose co-transporter-2 (SGLT-2) inhibitor empagliflozin can preserve beta-cell function and maintain glucose tolerance in women with recent GDM (ClinicalTrials.Gov NCT03215069), it is of interest that, in men and women with established T2DM and CVD, this medication can also reduce risks of atherosclerotic vascular disease and heart failure [22] (both of which are known to be elevated in women with a history of GDM [3, 9, 10]). Although both appropriate surveillance protocols and interventions remain to be established, the current data provide evidence to support the need for further studies of cardiovascular risk factor monitoring and modification in this patient population.

Strengths of this study are the repeated measurements of lipids and glycemic variables in 757,541 women over a sufficiently long duration of follow-up for CVD risk to manifest clinically. While the clinical basis for ordering these tests cannot be ascertained with these administrative data sources, the resultant potential selection bias likely identified slightly higher-risk non-GDM subgroups. Thus, the differences in cardiovascular risk factors between GDM and non-GDM subgroups observed in this study may actually be underestimated. In this context, it should be noted that comparison of women who were included versus those who were excluded because they had only one or fewer lab tests showed that the excluded women were slightly younger and more rural, though income levels and race were quite similar (data not shown). Another limitation is the absence of data on factors such as diet, physical activity, and adiposity (body mass index or waist circumference). However, the impact of this limitation on the current findings may be attenuated by two considerations. First, Dorans et al recently demonstrated that, compared to those with no history of GDM, women with previous GDM have higher A1c, fasting glucose, and LDL cholesterol, coupled with lower HDL cholesterol, after adjustment for age, race, education, smoking, diet, physical activity and waist circumference [23]. Second, it should be recognized that the cardiovascular impact of diet, physical activity, and adiposity is likely partially mediated through the glycemic and lipid variables under study. The downstream nature of these risk factors is evident in current thinking that metabolically healthy obesity generally requires transition of metabolically unhealthy obesity for the manifestation of incident CVD risk [24]. Accordingly, while the current study cannot account for all determinants of CVD risk in women with previous GDM, the risk mediated by these downstream glycemic and lipid variables remains valid and relevant. Indeed, the importance of this demonstration is further underscored by the fact women with GDM comprise a relatively young population that is identified early in the course of trajectories of worsening glycemia and dyslipidemia over time [16, 17, 25], such that early intervention could potentially modify their future risk of CVD. Another potential limitation of the study is that potential bias could be introduced to the traditional mediation analysis methodology used here by interaction between the mediator and the exposure or by confounding between the mediator and the outcome [26]. However, causal mediation analysis cannot be applied due to the absence of data on potentially important factors like physical activity or adiposity. Hence, the possibility of such bias tempers the interpretation of the study findings. Finally, due to the exploratory nature of the study, we did not correct for multiple tests of hypothesis. However, given the levels of statistical significance that were found, such correction would not substantially change the interpretation of the study findings.

## Conclusions

In conclusion, irrespective of whether they develop T2DM, women with a history of GDM have greater exposure to glycemia and dyslipidemia over time than that of their peers. This exposure holds clinical relevance because these adverse glycemic and lipid measures are mediators of their elevated risk of CVD, with the impact of lipids particularly apparent in those who do not develop diabetes. In identifying glycemia and dyslipidemia as determinants of CVD risk in women with previous GDM, these data provide potential targets for risk factor monitoring and ultimately early intervention towards the goal of primary prevention of CVD in this patient population.

## Supplementary Information

**Additional file 1: Table S1.** Correlation matrix between the biomarkers under consideration in the study.

## Data Availability

Restrictions apply to the availability of all data analyzed during this study to preserve patient confidentiality or because they were used under license. The corresponding author will, on request, detail the restrictions and any conditions under which access to some data may be provided.
