# Supplementary material for: Mediating effect of vascular risk factors underlying the link between gestational diabetes and cardiovascular disease
Source: BMC Med. 2022 Nov 4;20:389. doi: 10.1186/s12916-022-02581-0 (PMC9635213; doi:10.1186/s12916-022-02581-0)
Supplement: Supplementary file 1 — Additional file 1: Table S1. Correlation matrix between the biomarkers under consideration in the study. [file 12916_2022_2581_MOESM1_ESM.docx]

**Table S1:** Correlation matrix between the biomarkers under consideration in the study.

|  | **TChol** | **LDL** | **HDL** | **TG** | **A1c** |
| --- | --- | --- | --- | --- | --- |
| **Fast Gluc** | 0.03957 | 0.02098 | –0.21018 | 0.30919 | 0.82013 |
| **A1c** | 0.06049 | 0.05608 | –0.22691 | 0.28992 |  |
| **TG** | 0.34570 | 0.22682 | –0.43353 |  |  |
| **HDL** | 0.17871 | –0.12734 |  |  |  |
| **LDL** | 0.88970 |  |  |  |  |

Correlation statics determined using PROC CORR. Note that not all subjects had measurements of all of the cardiovascular risk factors. Thus, the above correlation table consists of pair-wise correlations wherein a given correlation coefficient is based on those subjects who had measurements of the two risk factors in question.
